# Lenalidomide-induced Pseudogout and Crowned Dens Syndrome

**DOI:** 10.31662/jmaj.2024-0028

**Published:** 2024-06-03

**Authors:** Kosuke Obama, Hana Yamamoto, Hirosaka Inoue

**Affiliations:** 1 Department of Hematology, Imakiire General Hospital, Kagoshima, Japan

**Keywords:** lenalidomide, pseudogout, crowned dens syndrome, myeloma

An 84-year-old male diagnosed with IgA myeloma was treated with lenalidomide. The disease was managed adequately; however, after 11 months, he experienced high-grade fever, swelling and pain in his right elbow, and severe posterior neck pain. A cloudy joint fluid was identified (Figure 1A), and the patient was diagnosed with pseudogout. Moreover, he was diagnosed with crowned dens syndrome based on cervical spine findings of computed tomography (Figure 1B). Discontinuation of lenalidomide and administration of nonsteroidal anti-inflammatory drugs reduced these symptoms. Nine months after the lenalidomide cessation, myeloma worsened, and readministration of lenalidomide led to generalized arthralgia and severe swelling of the left ankle joint; lenalidomide-induced arthritis occurrence was clinically confirmed. In Japan, 24, 8, and 1 cases of mild arthralgia, arthritis, and joint swelling, respectively, have been reported. Notably, some joint symptoms can be associated with lenalidomide-associated pseudogout.

**Figure 1.**
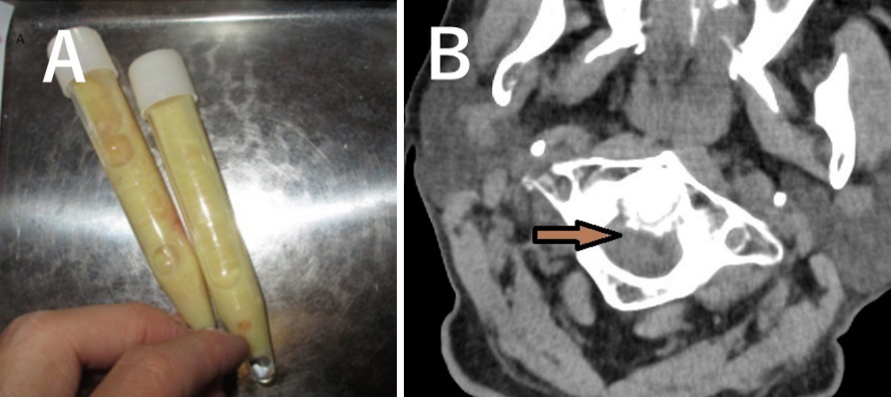
A: Arthrocentesis of the patient’s right elbow reveals a cloudy, sterile joint fluid with numerous calcium pyrophosphate crystals and leukocytes. B: Computed tomography indicates calcification around the axial vertebral dentate process.

## Article Information

### Conflicts of Interest

None

### Author Contributions

Kosuke Obama: planning and conducting research and writing the manuscript

Hana Yamamoto and Hirosaka Inoue: treatment coresponsibility

### ORCID iD

Kosuke Obama: 0000-0001-6717-1531

### Approval by Institutional Review Board (IRB)

Approval from the ethical board was not required.

### Informed Consent

Written informed consent was obtained from the patient by the corresponding author.

